# Strengthening Primary Care for Recognising and Treating Depression (SPiRiT-D): a study protocol for a cluster randomised controlled effectiveness-implementation trial of collaborative care for depression

**DOI:** 10.1186/s13063-026-09453-5

**Published:** 2026-01-28

**Authors:** Mehreen Riaz Faisal, Mujeeb Masud Bhatti, Simon Walker, Sheraz Ahmad Khan, Catherine E Hewitt, Fakiha Tus Salam, Faiza Aslam, Karen Coales, Mohammad Bilal Jawaid, Simon Gilbody, Najma Siddiqi

**Affiliations:** 1 Department of Health Sciences, University of York, York, UK; 2 Institute of Psychiatry, Rawalpindi Medical University, Rawalpindi, Pakistan; 3 Department of Applied Psychology, Riphah International University, Islamabad, Pakistan; 4 Centre for Health Economics, University of York, York, UK; 5 The Initiative, Islamabad, Pakistan; 6 SINA Health, Education and Welfare Trust, Karachi, Pakistan; 7 Hull York Medical School, York, UK; 8 Bradford District Care NHS Foundation Trust (BDCFT), Bradford, UK

**Keywords:** Collaborative care, Depression, Hybrid Type II, Implementation strategies

## Abstract

**Background:**

The effectiveness of collaborative care for treating depression in primary care has been well-established in high-income countries and, more recently, in a few trials in low- and middle-income countries (LMICs). However, evidence for its effectiveness, costs and how it can be implemented in ‘real-world’ settings within resource-constrained health systems in LMICs is currently limited. We aim to investigate the implementation, clinical and cost-effectiveness of a contextually adapted collaborative care model for depression in primary care clinics in Pakistan.

**Methods:**

A hybrid type-II effectiveness-implementation cluster randomised controlled trial with embedded process and economic evaluations will be conducted. Twenty-four primary care clinics located in socioeconomically disadvantaged areas of Karachi will be randomly allocated (1:1) using minimisation to either (i) a contextually adapted collaborative care model for depression supported by co-designed implementation strategies or (ii) optimised usual care (routine practice with additional depression screening and provision of information leaflets about depression for those screening positive). Participants aged 18 years or above, scoring ≥ 10 on the 9-item Patient Health Questionnaire (PHQ-9) and not under any active treatment for depression, will be recruited. The Reach, Effectiveness, Adoption, Implementation and Maintenance framework will guide our outcome evaluation. The primary clinical outcome will be depression severity, assessed using the PHQ-9 at 6 months after participant recruitment. The primary implementation outcome will be ‘reach’ (proportion calculated as the number of people who participated in depression treatment divided by those eligible for such treatment) using routine, clinic-level aggregated data at 6 months. The process evaluation will explore factors such as fidelity, acceptability and sustainability of collaborative care using a mixed-methods approach guided by the Consolidated Framework for Implementation Research. A within-trial economic evaluation will explore the cost-effectiveness of both collaborative care and implementation activities. Individual-level effectiveness outcomes will be analysed using mixed-effect linear regression; and clinic-level implementation outcomes using generalised linear regression. Trial data analysis will be based on an intention to treat principle.

**Discussion:**

If collaborative care is shown to be successfully implemented, clinically, and cost-effective, it will provide health and economic benefits for people with depression presenting in primary care. It will also be a means to strengthen primary care services through a trained workforce that can recognise and manage depression, improve information management systems, and promote evidence-based care.

**Trial registration:**

ISRCTN13462277 prospectively registered on 07 October 2024 10.1186/ISRCTN13462277.

**Supplementary Information:**

The online version contains supplementary material available at 10.1186/s13063-026-09453-5.

## Background

Depression is one of the most common of all mental disorders, affecting 280 million people globally, with a higher number of cases in women (170 million) compared to men (109 million) [1]. It significantly impacts the lives of those who are affected, their families and society [2]. The negative effects are far reaching, hindering access to educational, occupational, and economic opportunities [3]. The presence of depression in individuals with physical illness worsens outcomes for both conditions, resulting in earlier and higher mortality rates, as well as increases in healthcare costs [4, 5].

Treatment coverage for depression is poor in many parts of the world and this is particularly true for low- and middle-income countries (LMICs). Only 8% of individuals identified with depression receive mental health services in LMICs, compared to 33% in high-income countries (HICs) [6]. Many countries still lack the healthcare infrastructure, mental health policy, legislation, or adequate resources to deliver mental health programmes [7]. Pakistan is an LMIC and is the fifth most populated country in the world. Epidemiological evidence suggests a high rate of depression, 6% in the general population [8] surpassing the 5% global prevalence rate [9]; which may still be an underrepresentation of the actual prevalence. Despite underreporting, depression is now the second major cause of disability in Pakistan [4]. Tackling depression is, therefore, a high priority, especially in the wake of COVID-19, recent political and economic crises and natural disasters such as floods, resulting in the loss of lives, homes and economic opportunities, increasing the population’s vulnerability to mental disorders. Compounding the challenge, the current healthcare infrastructure, workforce and financing are significantly under-resourced to address the rising burden of mental disorders [10].

Training non-specialists in primary care to deliver evidence-based treatments for depression could be a possible solution. The World Health Organization (WHO) advocates for integrating low-cost mental health services in primary care and offers a range of guidance, training and supporting material to implement this in practice [11]. Among the various mental health care delivery models, the collaborative care (CC) model stands out as the most extensively tested and supported by robust evidence [12, 13]. CC is a low-cost, joined-up, integrated and patient-centred model of service delivery for physical and mental health, appropriate for primary care settings [14]. One of the notable aspects of the CC model is task sharing, in which non-specialists are trained to provide mental health care under supervision of mental health specialists. Other key components of CC include (i) population-level identification of depression; (ii) provision and adjustment of evidence-based care; (iii) tracking treatment outcomes and (iv) case review meetings [14, 15].

Evidence-based treatment for depression includes the delivery of psychotherapy and/or antidepressants [16, 17]. There is considerable evidence that non-specialist healthcare workers can be trained to deliver brief but effective psychological treatments for common mental disorders [18, 19]. Mild to moderate cases can be managed by non-specialists, while severe or treatment-resistant cases are referred to mental health specialists, this is known as the ‘stepped-care’ approach.

Behavioural activation (BA) is a relatively simple and flexible psychological therapy which has been proven to be effective for the treatment of depression [20], and can be delivered using a CC model. It is recognised for its parsimony, portability across cultures and efficiency of training [21–23]. It works by identifying activities that the person with depression may be avoiding due to loss of motivation or feeling overwhelmed as a consequence of being depressed. The focus of BA is to break the cycle of depression and to re-engage people with stable and diverse sources of positive reinforcement from the outside world and to develop depression management strategies for future use [22].

The clinical and cost-effectiveness of the CC model has been tested in many HICs [24] with evidence indicating CC leads to significant improvements in depression and anxiety, while also reducing associated healthcare costs [25–27]. A recent systematic review also reported effectiveness of CC in LMICs for improving depression outcomes at short (3 months), medium (6 months), and long-term (12 months) follow-ups [13]. However, this evidence is limited, particularly for South Asian countries, with only three trials conducted [28–30].

Despite the known clinical effectiveness of CC, there is a considerable evidence-implementation gap [31, 32], which is more pronounced in resource-limited settings in LMICs, where the implementation of evidence-based practices is not well-understood [33]. Evaluating the implementation and effectiveness of CC in diverse contexts, particularly in LMICs, could offer valuable insights. We therefore aim to evaluate the implementation, clinical effectiveness and cost-effectiveness of CC, using co-designed implementation strategies, for improving depression outcomes in primary care clinics in Pakistan.

## Objectives

Our specific objectives are to:

- Evaluate the implementation, clinical effectiveness, and cost-effectiveness of the CC model for depression compared to optimised usual care.
- Assess the fidelity, quality and acceptability of the CC model and identify the mechanisms of change and contextual factors associated with implementation outcomes.

## Methods

### Study design

The study design is an effectiveness-implementation hybrid type-II trial with cluster randomisation to evaluate CC in primary care clinics in Karachi, Pakistan. A mix of qualitative and quantitative methods will be used to collect data on clinical effectiveness, and implementation outcomes. An embedded process evaluation will help to understand implementation processes, and an economic evaluation will assess cost-effectiveness. Trial registration data is presented in Table 1. The SPIRIT reporting checklist is included as additional information [34].

**Table 1:** Trial registration data

Data category	Information
Primary registry and trial identifying number	ISRCTN ISRCTN13462277
Date of registration in primary registry	October 07, 2024
Source(s) of monetary or material support	NIHR under Global Health Research Centre programme (NIHR203248)
Primary sponsor	The University of York, UK
Contact for public inquiries	Mujeeb Bhatti, PhD
Contact for scientific queries	Mehreen Faisal, PhD
Public title	Improving services for identifying and treating depression in primary healthcare clinics
Scientific title	Strengthening Primary care for Recognising and Treating Depression (SPiRiT-D)
Countries of recruitment	Pakistan
Health condition(s) or problem(s) studied	Depression
Intervention(s)	The intervention involves implementing a collaborative care model for depression in primary healthcare units. This model includes five key components: systematic identification of depression, provision and adjustment of evidence-based care (both antidepressants and brief behavioural treatment), tracking treatment outcomes, and weekly case review and supervision meetings. A collaborative care model for identifying and managing depression will be introduced in all intervention clinics using various implementation strategies
Key inclusion and exclusion criteria	Inclusion criteria: 1. Attending primary care clinics, 2. Aged 18 years or above, 3. Score on PHQ-9 ≥ 10 Exclusion criteria: 1. Participants already receiving any kind of treatment for depression, 2. Participants lacking the capacity to provide informed consent, or not willing to consent
Study type	Effectiveness-implementation hybrid type II cluster randomised controlled trial with an embedded economic and a mixed-methods process evaluation
Date of first enrolment	June 02, 2025
Target sample size	750
Recruitment status	Recruiting
Primary outcome(s)	1. Effectiveness: Depression severity (mean change) measured using Patient Health Questionnaire-9 (PHQ-9) at the 6-month timepoint 2. Implementation: Reach of depression treatment (RT) using RT = st/ne, where ‘st’ represents the number of individuals who participated in the treatment for depression and ‘ne’ represents the number of people eligible for such treatment at 6 months
Key secondary outcome(s)	The following secondary outcomes related to clinical effectiveness will be measured at the participant level during follow-ups at 3, 6, and 12 months, unless otherwise specified below: 1. Body mass index (BMI) calculated using the formula: BMI = weight (kg)/height (m^2^), where height is measured in metres and weight is measured in kilograms 2. Blood pressure measured using an automated blood pressure instrument 3. Waist circumference measured in centimetres 4. Depression severity measured using PHQ-9 at 3 and 12 months 5. Depression caseness measured using PHQ-9 6. Anxiety caseness and severity measured using Generalised Anxiety Disorder Questionnaire (GAD-7) 7. Quality of life measured using Euroqol’s instrument EQ-5D-5L 8. Functional impairment measured using the WHO Disability Assessment Schedule (WHODAS 2.0) at 6 and 12 months 9. Healthcare resource measured using modified Client Service Receipt Inventory (CSRI) at 6 and 12 months 10. Adverse events measured using a modified adverse event The following clinic-level aggregated secondary outcomes related to implementation effectiveness will be calculated at 3, 6, and 12 months follow-up timepoints, unless otherwise specified below: 11. Reach of screening (RS) calculated using the formula RS = sd/nc, where ‘sd’ represents the number of individuals screened for depression and ‘nc’ denotes the total number of individuals attending the clinics 12. Reach of depression treatment (RT) calculated using RT = st/ne, where ‘st’ represents the number of individuals who participated in the treatment for depression and ‘ne’ represents the number of people eligible for such treatment at 3 and 12 months 13. Adoption (AD) calculated using AD = r/nr, where ‘r’ denotes the number of people referred for the treatment of depression and ‘nr’ denotes the number of people eligible for referrals at 3, and 6 months 14. Treatment enrolment (TE) calculated using TE = te/ne, where ‘te’ represents the number of people enrolled in the treatment of depression and ‘ne’ represents the number of people eligible for such treatment 15. Treatment completion (TC) calculated using TC = tc/te, where ‘tc’ represents the number of people completed the depression treatment and ‘te’ the number of people enrolled in such treatment 16. Treatment drop (TD) calculated using TD = td/te, where ‘td’ represents the number of people dropped out of the depression treatment and ‘te’ the number of people enrolled in such treatment 17. Sustainability calculated using change in percentage from baseline to follow-up time-points in reach, adoption, treatment enrolment, completion, and dropout 18. Equity by comparing reach, adoption, treatment enrolment, completion, and dropout across different levels of socioeconomic status, gender, and ethnicity

### Study setting

The study will be based in primary care clinics of SINA Health, Education and Welfare Trust [35], which is a not-for-profit trust that has established 38 primary care clinics in the most impoverished areas of Karachi, Pakistan. SINA offers comprehensive primary healthcare services, including a limited mental health programme, providing treatment to around 1.5 million people annually. SINA clinics serve the most economically deprived populations, providing healthcare at a minimal cost or free of charge, for those assessed to be entitled for welfare. They are relatively well-resourced, including the use of electronic medical records (EMR) in the majority of the clinics. SINA clinics are categorised into three main types of facilities based on the number and type of staff available, and the services they offer: (i) availability of > 2 primary care physicians (PCP) and a lay counsellor (*N* = 10), (ii) those with > 2 PCP but no lay counsellor (*n* = 17), and (iii) those staffed with only one PCP (*n* = 11). Lay counsellors are non-specialist mental health care providers without any formal education in mental health. They receive training to screen for common mental disorders, including depression, using simple tools such as Patient Health Questionnaire-9 (PHQ-9), and are trained to deliver brief psychological therapy suited for non-specialists. They also refer patients to mental health specialists when more intensive care is needed. Their work is carried out under the supervision of qualified mental health professionals [36]. Presently, only a few clinics offer antidepressant medications.

### Eligibility criteria

All patients ≥ 18 years attending the trial primary care facilities with depression symptoms identified following screening with PHQ-9 having a score of ≥ 10, will be eligible to take part in the study. Those already receiving any psychological therapy, lacking the capacity to provide informed consent, or not willing to participate will be excluded.

The PHQ-9 includes nine items and is scored on a scale from 0 to 3 according to the frequency of experiencing the symptom during the last 2 weeks, ranging from ‘not at all’ to ‘nearly every day.’ The total score ranges from 0 to 27. Scores between 0 and 4 indicate absence of depressive symptoms; 5–9 mild depressive symptoms; 10–14 moderate depressive symptoms; 15–19 moderately severe depressive symptoms; and 20–27 severe depressive symptoms [37, 38].

### Randomisation

**Fig. 1:**
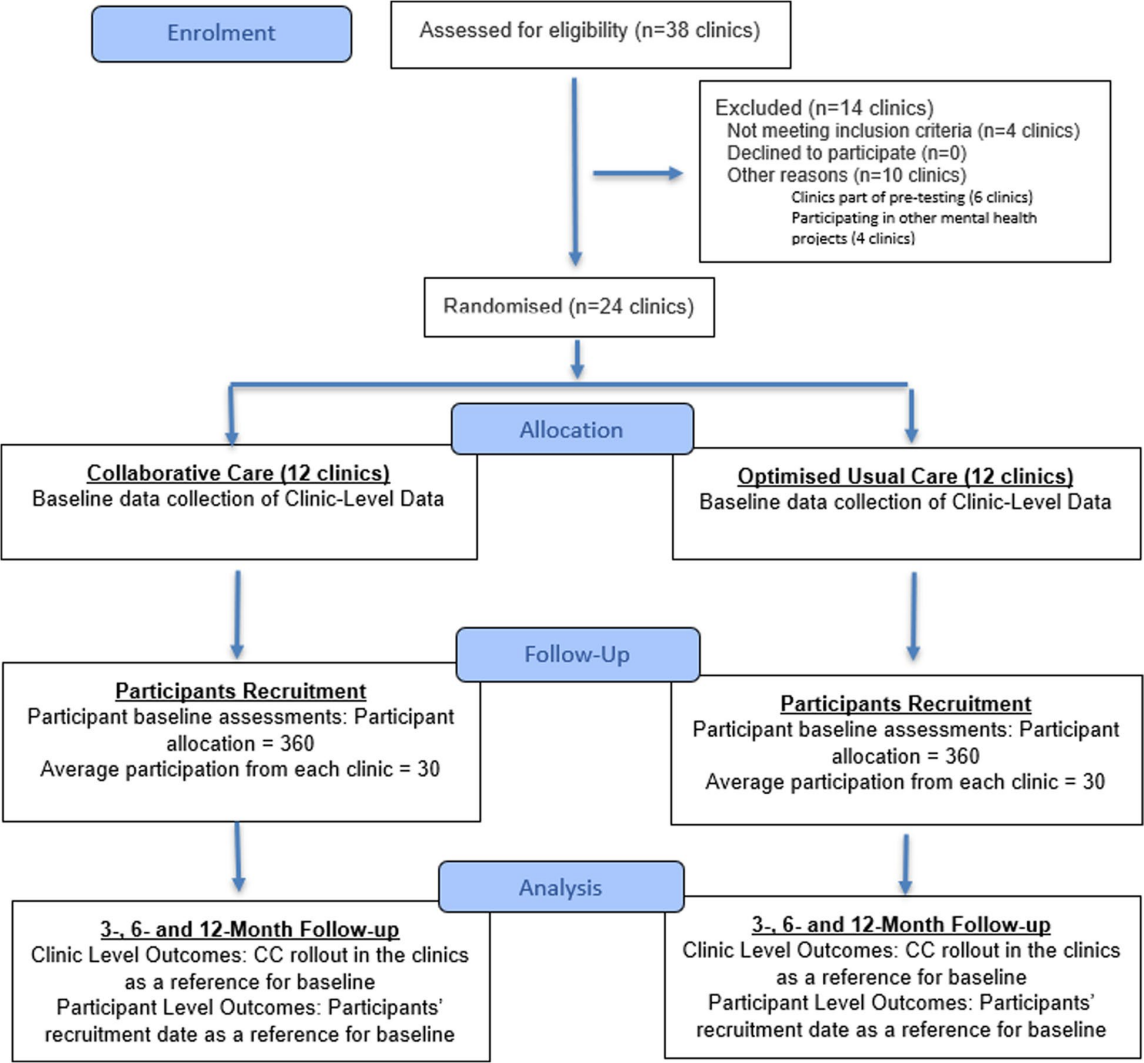
CONSORT flow diagram

Randomisation will be at the primary care clinic level which will be the ‘cluster’. Minimisation technique will be used to achieve balance on three characteristics: (i) clinic size or average attendance per month (small < 1000, medium 1001–3000, large > 3000), (ii) availability of EMR (present/absent) and (iii) counsellors (previously present/absent). Once all clinics are recruited, randomisation of the clusters will be undertaken by the York Trials Unit using minimPy [39] (See Fig. 1).

### Recruitment

Recruitment of patients will be based on a two-stage screening process. All patients attending the trial clinics will be screened by trained PCPs for depressive symptoms initially using the PHQ-2 (a brief version of PHQ-9). Patients screening positive on PHQ-2 (score ≥ 3) will be referred to the counsellors for assessment using the PHQ-9. This will be implemented as part of routine practice in both the intervention and control clinics. To balance the intervention and control clinics except for the intervention, we plan to recruit counsellors for those trial clinics which do not have counsellors present currently.

In the intervention clinics as part of the CC model, patients who screen positive on PHQ-9 (score 10 or above) will be offered BA therapy, antidepressants, or their combination as per treatment protocol. In control clinics, optimised usual care (OUC) will be offered, including information leaflets and routine psychological treatment. Patients who fulfil the trial inclusion criteria will be approached by the researcher present, who will provide the study information sheet in the local language. Written informed consent will be obtained before trial enrolment. Where there are issues of literacy, the information will be read out by researchers and understanding checked; consent will be recorded via thumbprint signatures. Potential participants will be informed that their decision to take part in the study will not affect their entitlement to usual services and that they are free to withdraw from the study at any stage without having to provide a reason. If participants are willing to provide reasons for withdrawal, this will be recorded in the change of status form. There are no cross-overs intended as part of the trial design, and cross-overs of the trial participants between the intervention and control clinics are less likely to happen because of the separate catchment areas that the different clinics serve. However, the central electronic records system will help identify any potential cross-overs, in the unlikely case of it happening.

### Baseline assessments

All consenting participants will undergo baseline assessment at recruitment or within 1 week through a questionnaire and clinical assessments to record demographics (age, sex, ethnicity) [40], education, language(s), employment status, socioeconomic status [41], household expenditures, productivity loss, history of depression, chronic physical health conditions (diabetes, cardiovascular disease, lung disease), other mental illness, current depression [37] and anxiety severity [42], quality of life [43], functional impairment [44], healthcare resource use [45], body mass index (BMI), blood pressure (BP) and blood tests (haemoglobin, glycosylated haemoglobin (HBA1c)) (see details of tools in outcomes section).

### Blinding

Blinding to the intervention will not be possible for study participants, or those involved in its delivery, due to its nature. We will endeavour to keep researchers involved in recruitment, baseline and outcome data collection to remain blinded to the allocation of clinics. However, there is a risk that this cannot be maintained if there are visible changes to clinics because of implementation strategies. We will check the maintenance of blinding during and at the end of the trial.

### Interventions

Our main intervention inputs are (i) the CC model and (ii) implementation strategies to support contextually adapted CC model for depression in the intervention clinics.

The implementation team (IT) led by two implementation champions from SINA, including clinic staff (PCPs, lay counsellors, clinic supervisors), SINA management (programme manager, mental health programme officer, quality assurance manager, and assistant manager of data analytics), and research team members, helped devise implementation strategies and finalise components of the CC model during the co-design stage previously, through involvement of the service users, carers, and community members. The implementation strategies were then refined through rapid testing and feedback from staff and service users across six test clinics (details will be reported in a separate publication).

#### Collaborative care model

**Fig. 2:**
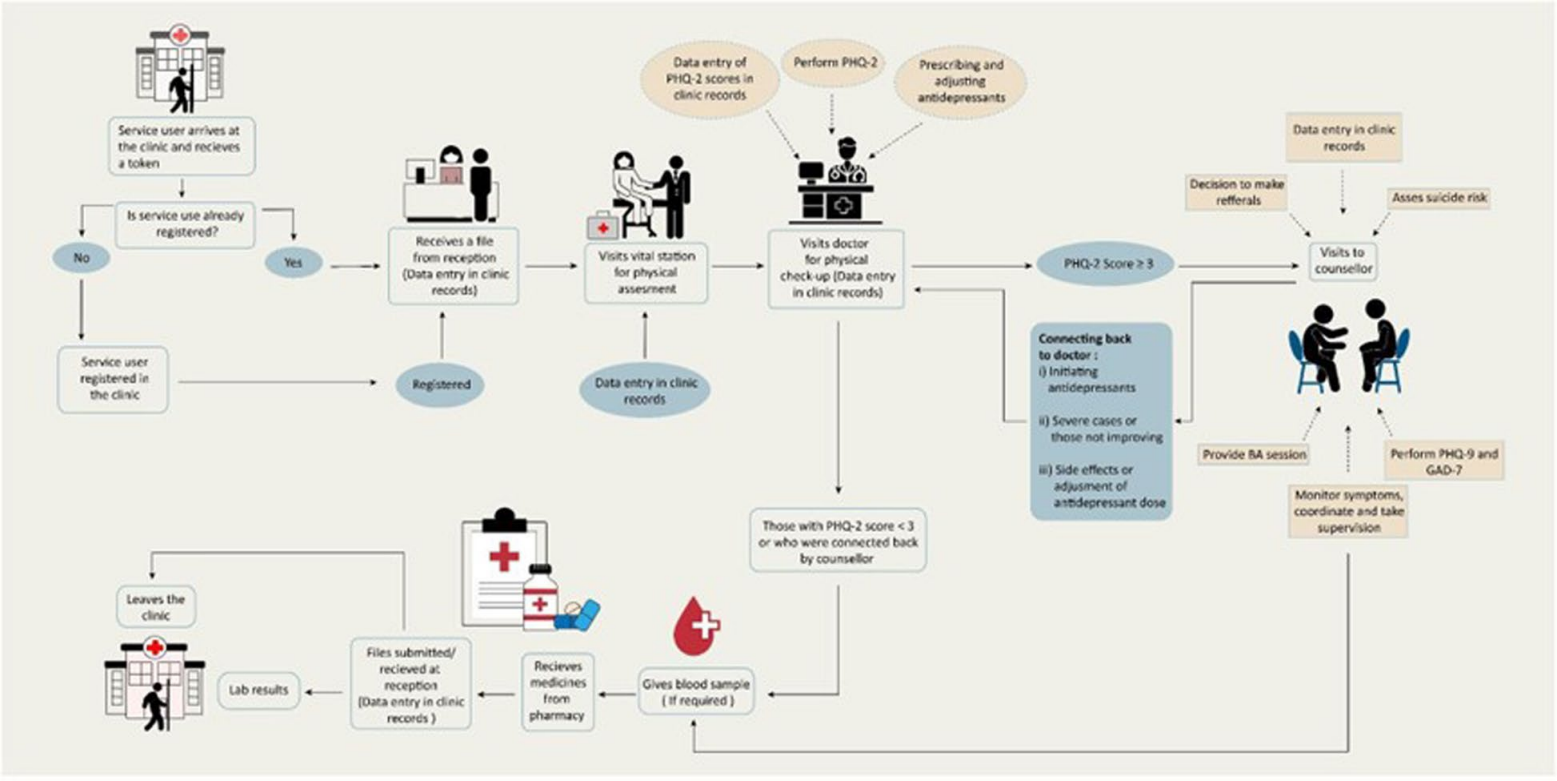
Collaborative care model implementation

The CC components co-designed considering the SINA context include the following: (i) two-stepped systematic identification of depression for all people aged ≥ 18. In the first stage, PCPs will screen patients using the PHQ-2, those scoring ≥ 3 will be referred to the lay counsellor. In the second stage, the counsellors will use the PHQ-9 to identify depression; (ii) counsellors will offer BA [38] therapy to patients scoring ≥ 10 on the PHQ-9. If a patient does not respond or prefers antidepressants, counsellors will refer the patient to the PCP to initiate antidepressant treatment; (iii) counsellors will track and monitor depression (e.g. the severity of depression symptoms) and process measures (e.g. number of contacts, care provided, and review with the mental health specialist) for all patients currently receiving care; (iv) counsellors and PCPs will collaboratively adjust care, using a stepped care approach such as augmenting BA with medication or changing the medication dose until care goals (improvement or remission of depression) are achieved; and (v) counsellors and PCPs (if required) will attend weekly supervision and case review meetings with the mental health specialists (See Fig. 2).

#### Implementation strategies

The co-designed implementation strategies based on Expert Recommendations for Implementing Change (ERIC) [46, 47], to support the implementation of contextually adapted CC are provided in Table 2.

**Table 2:** List of implementation strategies to be used as part of the SPIRIT-D trial

Implementation strategies*	Description
**Domain: Train and educate stakeholders**	
**Develop educational materials**	Educational materials will be developed including guidelines for identifying depression using PHQ-2 and PHQ-9 for screening; an information leaflet for the patients about depression and a list of nearby facilities for depression care; BA therapy manual and patient guide; a desktop guide for the physicians on depression management and antidepressant prescription
**Distribute educational materials**	Clinics will be provided with the following educational materials: guidelines for identifying depression using tools like PHQ-9, a list of nearby referral facilities to help service users access appropriate care, BA materials (facilitator and patient guide), a desktop guide for depression management including prescribing antidepressants, and a risk assessment protocol for suicide
**Conduct ongoing training**	The research team, with input from the implementation team, will develop or modify training packages on (i) identification of depression, for physicians and lay counsellors, (ii) BA training for counsellors and supervisors, and additional training for BA supervisors, (iii) depression management including prescription of antidepressants for the physicians, (vi) data management including maintaining the confidentiality of medical/clinical records and (v) the CC model including aspects of care coordination and the use of population registry utilising a bespoke spreadsheet or paper-based registers
**Make training dynamic**	The training will make use of interactive methods such as role-plays, discussions and experience sharing
**Use train-the-trainer strategies**	Our training method will use a train-the-trainer model for specific training where possible. Psychologists are already based at the head office/central location in SINA. The research team, experienced in identifying depression, delivering and supervising BA, will train the psychologists in (i) identifying depression, (ii) delivering BA, (iii) supervising BA, and (iv) training others in BA. The psychologists will further take on the role of BA trainers and actively lead the training for counsellors on BA and mental health specialists (psychiatrists) on BA supervision, during the rollout of CC. However, to support those newly trained trainers/supervisors, experienced BA trainers and supervisors who are a part of the research team will stay in touch remotely. PCPs will be trained by the psychiatrists on depression management including prescription of antidepressants
**Domain: Provide interactive assistance**	
**Facilitation**	The implementation will follow a guide developed for this purpose, following five stages of implementation: (i) exploration, (ii) preparing for practice change, (iii) training, (iv) launching care, and (v) sustainability. To develop a shared understanding of the guide, implementation, and research team members will meet and set agendas and plans before each implementation stage
**Provide local technical assistance**	Technical assistance will be taken for several matters relating to implementation from the SINA head office including but not limited to (i) identifying and hiring staff and developing/revising job descriptions, (ii) arranging and scheduling training, (iii) calling meetings to introduce change in practice, (iv) development and implementation of registry and tracking tools, (iv) any changes to consumer fees or other financial decisions, and (iv) scheduling case review and supervision meetings
**Provide clinical supervision**	Mental health specialists will include psychiatrists to provide consultation and supervision to counsellors and physicians on case reviews and caseload management, and BA supervision. A preliminary supervision format was developed from the co-design workshops and implementation team meetings. BA supervision and case review meetings are to be conducted remotely or in-person in a group format, including the counsellor, physician (if needed), and the psychiatrists, who can join via phone, Zoom, or other encrypted services. Additionally there will be drop-in sessions available and a Whatsgroup established, separate for counsellors, and physicians to facilitate contact with the psychiatrists if needed, at indicated specific times
**Domain: Support clinicians**	
**Remind clinicians**	Workflows and flowcharts will be developed with laminated copies pasted over the physician’s desks to act as a reminder as well as a quick reference guide
**Revise professional roles**	SINA head office will hire new staff or revise the roles of the existing staff for clinics that are randomised to intervention group. Lay counsellors (where not already available), and psychiatrists will be hired. However, the roles of physicians and psychologists currently employed by SINA will be revised. Lay counsellors will work full-time, 6 days a week, supporting and coordinating mental health care, delivering BA, monitoring treatment response, and scheduling weekly care reviews and supervision meetings. Two part-time psychiatrists (approximately 3 h per week) will provide consultations to 6 clinics each, advising on medications and treatment strategies, and participating in weekly case review meetings. The physicians in all the clinics will take on additional duties, including identification of depression, prescribing antidepressants as needed, updating depression scores and clinical observations in clinic records, referring patients to counsellors, adjusting antidepressant doses through systematic tracking, and attending case review meetings when required. Two psychologists from SINA will serve as BA master trainers, ensuring delivery of BA training to new lay counsellors recruited for the intervention sites. Job descriptions for all those with revised roles or hired will be updated
**Facilitate relay of clinical data to providers**	The physicians will collaborate with lay counsellors via shared access to patient records and participate in weekly case reviews
**Domain: Change infrastructure**	
**Change physical structure and equipment**	The physical infrastructure and resources needed to implement CC will be identified and arranged, including dedicated rooms for counsellors, necessary furniture, tools for tracking and monitoring routine data, supply of antidepressants
**Change record systems**	A paper-based record system through a screening record register will be implemented to keep a record of the number of patients screened and also to monitor the screening interval for individual patients. In addition, the EMR interface (where it is available) will also be edited if possible, to allow entry of patient screening records

^*^Based on Expert Recommendations for Implementing Change (ERIC) implementation strategies

**Fig. 3:**
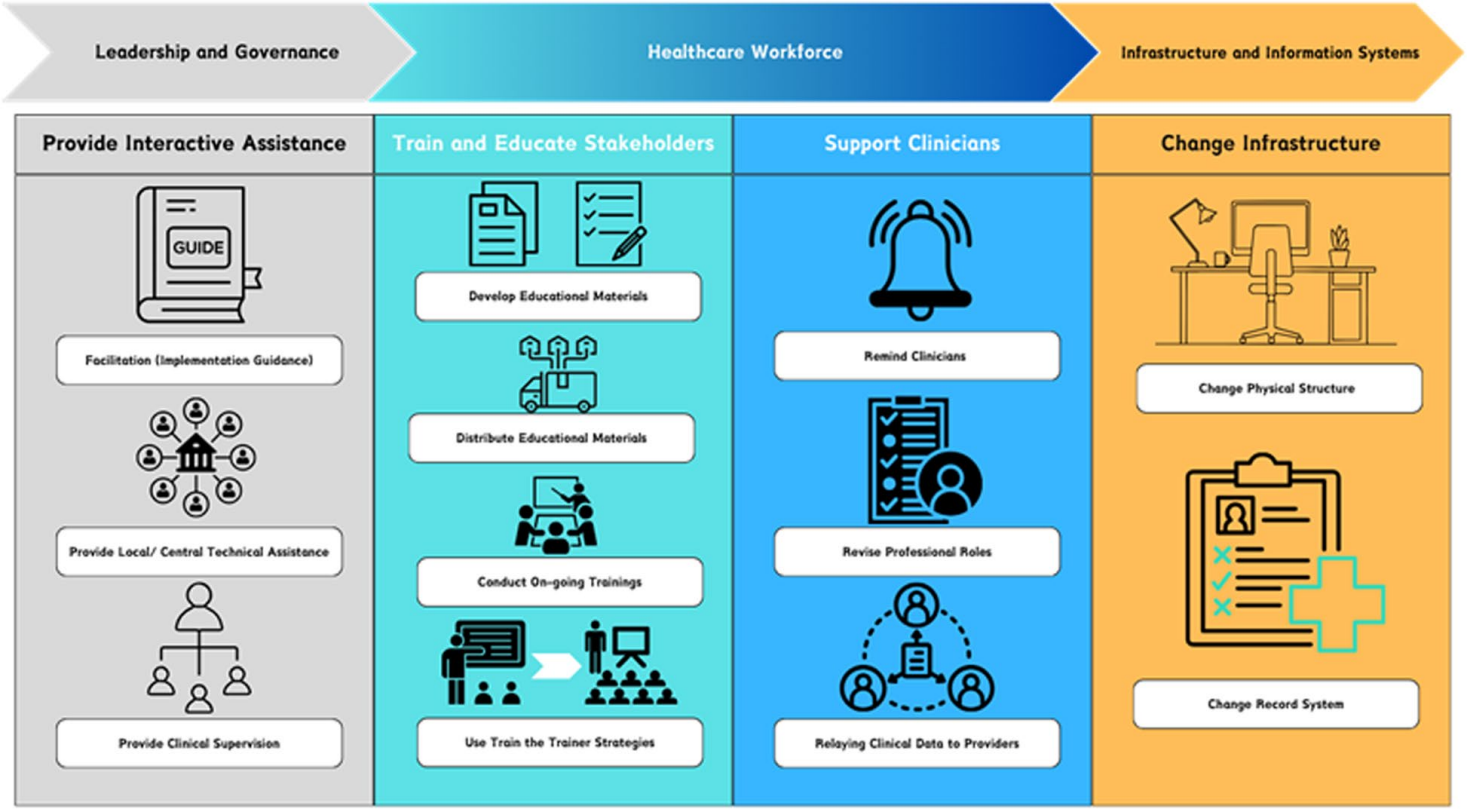
Illustration of implementation strategies

Figure 3 presents a visual presentation of our implementation strategies and how they are linked to different components of healthcare systems.

### Optimised usual care

Clinics assigned to the OUC will follow the usual care pathway with some adjustments. The existing SINA care pathway for mental health varies depending on availability of the lay counsellor. If a counsellor is present, PCPs refer patients with distress symptoms for further evaluation, using tools like PHQ-4, PHQ-9, GAD-7 [42], and suicide risk assessment. If depression or anxiety is detected, counselling therapy is provided. In clinics without a counsellor, there is no formal system for identification of mental illness. As part of the OUC, we will introduce following changes: (i) all physicians will administer the PHQ-2 screening for depression to all patients aged ≥ 18, and if the PHQ-2 score is ≥ 3, physicians will refer patients to the counsellor and provide a leaflet with information about depression symptoms, treatment options, and nearby free mental health services and (ii) counsellors will be hired for clinics where there is not one available currently, who will screen patients using PHQ-9 and provide the usual care counselling therapy for those scoring ≥ 10.

### Outcomes

Outcomes in this trial are guided by the Reach, Effectiveness, Adoption, Implementation and Maintenance (RE-AIM) framework [48]. We will assess individual-level outcomes (for consenting trial participants) and clinic-level outcomes from aggregated, anonymised, routinely collected data in clinics (Table 3). For individual-level data, participant recruitment date will be used as a reference point for follow-ups, whereas reference point for clinical outcomes will be the CC rollout in the clinics. Table 3 shows the planned schedule of enrolment, interventions, and assessments.

**Table 3:** Enrolment, interventions, and assessments schedule

	**Study period**				
	**Enrolment**	**Baseline**	**Post-allocation**		**Close-out**
**Timepoints**	** − 7 days**	**0 days**	**3 months**	**6 months**	**12 months**
**ENROLMENT**					
PHQ-2 screening by physicians	X				
Eligibility screening by researcher (PHQ-9; age and current treatment)	X				
Informed consent	X				
Allocation	X				
**INTERVENTIONS**					
Collaborative Care	■				
Optimised Usual Care	■				
**ASSESSMENTS**					
**Individual level data—those consenting to trial (starting/baseline point – date of participant recruitment)**					
Demographics		X			
Education, employment status and language		X			
Socioeconomic status (SES)		X			
Blood tests (haemoglobin, glycosylated haemoglobin (HBA1c)		X			
BMI, BP and waist circumference		X	X	X	X
**Effectiveness**					
Depression severity (PHQ-9)				X	
Depression caseness (PHQ-9)		X	X	X	X
Anxiety severity and caseness (GAD-7)		X	X	X	X
Quality of life (EQ-5D-5L)		X	X	X	X
Functional Impairment (WHODAS 2.0)		X		X	X
Healthcare resource (Modified CSRI)		X		X	X
Economic outcomes		X		X	X
Adverse events			X	X	X
**Clinics level data—aggregated data (starting point/baseline – after setting up CC in clinics)**					
**Reach**					
Receipt of treatment for depression				X	
Depression screening		X	X	X	X
**Adoption**					
Referral rate			X	X	
Supervisions			X	X	
**Implementation**					
BA completion rate		X	X	X	X
BA dropout rate		X	X	X	X
Antidepressants dispense rate			X	X	X
Antidepressants			X	X	X
**Maintenance**					
Sustainability			X	X	X
Equity			X	X	X

#### Primary outcome—effectiveness

The primary outcome is the depression severity measured at 6 months by estimating the mean change in PHQ-9 depression scores from baseline to 6 months and comparing across allocation groups [37, 38].

#### Secondary outcomes—effectiveness

1. The prevalence of case-level depression by allocation groups will be measured using PHQ-9 scores; a score < 10 indicates recovery, while ≥ 10 indicates case-level depression [37, 49, 50].
2. The severity of anxiety will be measured by the mean change in GAD-7 scores from baseline to follow-up time points, across allocation groups. We will also assess the caseness of anxiety by allocation groups, using a GAD-7 score of ≥ 6 to indicate the clinical case-level of anxiety [38, 42].
3. The mean change in Health Related Quality of Life (HRQoL) from baseline to follow-up time points, by allocation groups, will be measured using the EuroQol Five-Dimensional Scale, five levels (EQ-5D-5L) instrument [43].
4. The severity of functional impairment will be measured by the mean change from baseline to follow-up time points by allocation groups using the World Health Organization Disability Assessment Schedule (WHODAS 2.0) [44].
5. Anthropometric measures include BMI, BP, and waist circumference [51].
6. Healthcare resource use will be assessed using a modified Client Service Receipt Inventory (CSRI). We will collect data covering several aspects including (i) outpatient and inpatient visits/admissions, (ii) medical tests, (iii) medicines, and (iv) details of specialities consulted. Costs will include total healthcare and out-of-pocket costs, including intervention costs [45].
7. Economic outcomes such as household expenditures and assets, productivity loss, and catastrophic healthcare costs will also be collected. This will be collected using the International Wealth Index (IWI) and some items will be developed purposefully to capture this detail [41].
8. Serious adverse events will be recorded and reported immediately, while adverse events will be recorded in follow-up.

Depression, anxiety, quality of life, anthropometric measures, and adverse events will be assessed at 3, 6, and 12 months. All other outcomes will be assessed at 6- and 12-month follow-up.

#### Primary outcome—implementation

Our primary outcome is the reach of depression treatment, measured by calculating the proportion of eligible individuals (score on PHQ-9 ≥ 10) who participate in treatment for depression (having ≥ two contacts with healthcare staff) 6 months after the CC rollout in the clinics, i.e. when clinic staff start practising CC in their clinics, referred to as the ‘start date’.

#### Secondary outcomes—implementation

Secondary implementation outcomes include:

1. The reach of screening as the percentage of people attending clinics who participated in screening for depression (PHQ-2 and PHQ-9) at least once.
2. Adoption of the intervention will be determined at 3 and 6 months through referral rates (number of patients referred for treatment divided by number of eligible patients). We will consider referrals, including within primary care settings and to specialised facilities if required. We will track the number of healthcare staff invited to training, those who attend, and the average number of supervision or case review meetings attended by staff each month. These metrics will be assessed in the 3rd and 6th month follow-up after the start date.
3. The implementation of the CC components will be assessed using treatment engagement and dropout rates. We will determine the enrollment, completion, and dropout rates for psychotherapy (BA therapy in intervention clinics and any psychotherapy delivered in control clinics). Individuals who attend ≥ 2 sessions will be categorised as ‘engaged with BA/any psychotherapy’, whereas those attending < 2 sessions will be considered dropouts. We will also evaluate the prescription and adherence rates of antidepressants using the total number of antidepressants prescribed and the participants who complete their prescribed course of antidepressant medication captured through patient self-reporting.
4. Sustainability will be assessed by comparing reach, BA completion and dropout, antidepressant prescription, and compliance rate over time.
5. Equity in the treatment is the just distribution of treatment services, ensuring the participation of all individuals regardless of their socioeconomic status (SES) and demographic backgrounds. To ascertain equity, we will compare implementation outcomes mentioned above by SES and demographics (age, sex, ethnicity, education level).

Unless otherwise stated above, all outcomes will be recorded and compared across allocation groups, where feasible at all time points (3, 6, and 12 months after the start date).

### Sample size

We calculated our sample size based on a recent meta-analysis of CC [13] with a pooled standardised effect size of − 0.51 (95% confidence interval (CI) − 0.80 to − 0.23) for depression outcomes measured up to 6 months. Using an estimate closer to the lower bound, i.e. − 0.35, which is approximately equivalent to 1.4 points on PHQ-9 (assuming SD = 4), we will require 720 participants from 24 clinics (~ 30 participants per clinic) and need to screen approximately 12,000 (assuming 10% depression prevalence, 60% consent rate, 20% attrition, − 0.35 standardised effect, 90% power, 5% alpha, 0.03 intraclass correlation coefficient and design effect of 1.69) to evaluate the clinical effectiveness of CC for depression.

### Statistical methods

Statistical analysis will be conducted using STATA version 18 (StataCorp, College Station, TX). All statistical tests will use 5% significance levels and 95% confidence intervals. Descriptive statistics (mean, standard deviation, counts, and percentage) shall be used to summarise both effectiveness and implementation outcomes at baseline and 3-, 6-, and 12-month follow-up time points by allocation groups. Assumptions of all parametric tests (such as normal distribution) where applicable and the presence of missing data at random will be evaluated; if not met, appropriate transformation or multiple imputation methods will be considered.

A mixed effects linear regression model will be used with a PHQ-9 score at 6 months as the outcome for the primary effectiveness endpoint. We will use the PHQ-9 baseline score and other important baseline variables as fixed effects and clinics as random effects to account for the clustered nature of the data. The same models (or logistic mixed effects model in case of binary outcome) will be used for other individual-level secondary variables, where applicable. All the analyses will be based on the intention-to-treat principle.

On the other hand, generalised linear model with a log link function and binomial distribution will be used for ‘reach’ (participants who received treatment divided by those eligible for treatment) as an aggregated implementation outcome to compare allocation groups while using some important clinic-level factors as covariates such as availability of EMR (present vs absent) and average attendance per month (small < 1000, medium 1001–3000, large > 3000). All other clinic-level data will be analysed using the same model, where comparisons across groups are applicable. Some clinic-level data captured only in the intervention group, such as adoption, will be summarised using descriptive statistics.

### Process evaluation

A mixed methods process evaluation embedded within the trial will be conducted to explore the implementation outcomes or determinants relevant to CC by capturing details on factors including:

I. Fidelity, reach, acceptability, appropriateness, and sustainability.
II. Barriers and facilitators to implementation.
III. Mechanism of impact.
IV. Scale-up and transferability.

**Fig. 4:**
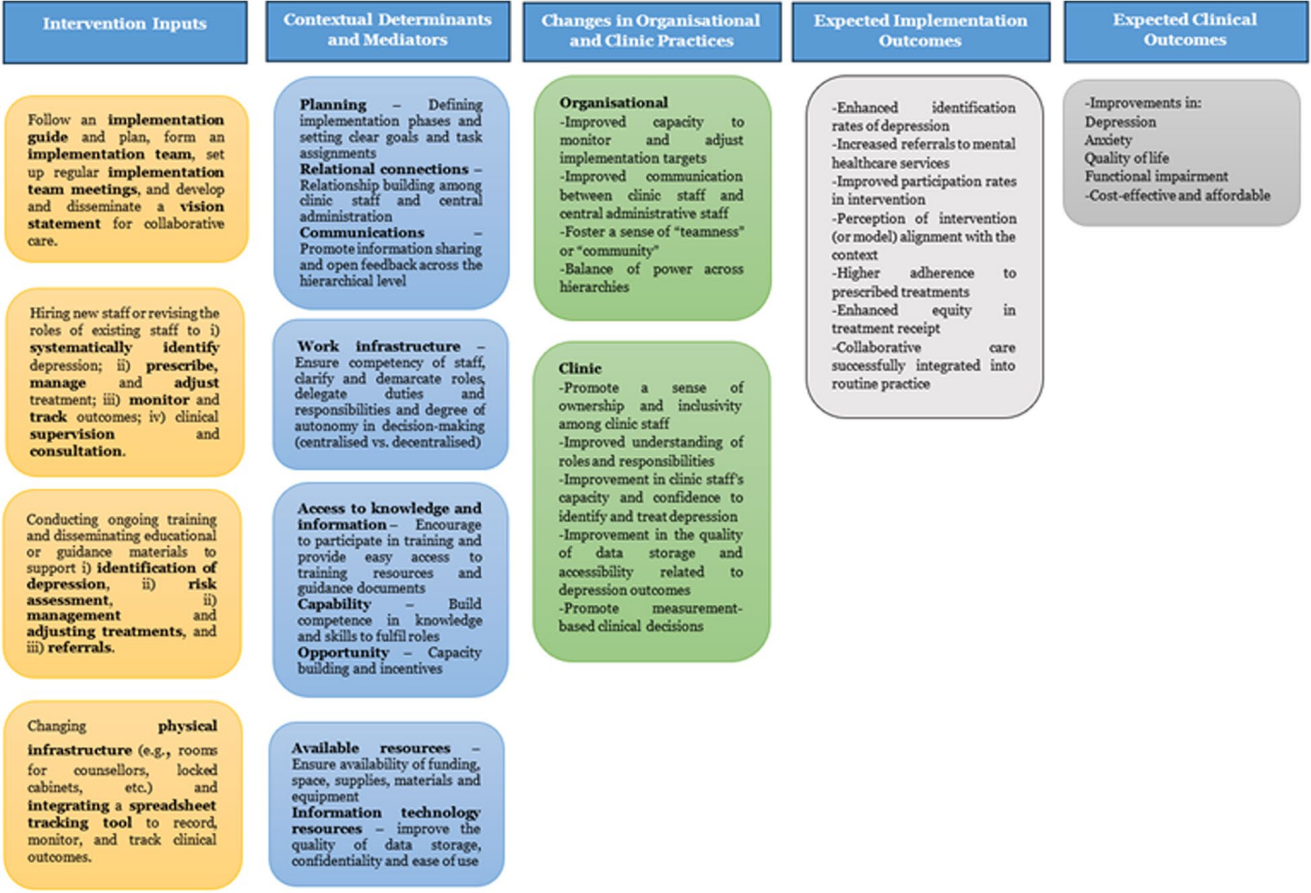
Logic model for implementation of collaborative care

Process evaluation will be driven by the CFIR [52]. CFIR is a comprehensive, structured, and widely used implementation framework that helps identify factors influencing the implementation of interventions in healthcare settings [53]. It comprises five major domains: (i) intervention characteristics, (ii) outer setting, (iii) inner setting, (iv) characteristics of individuals involved, and (v) implementation process. Based on the prior engagements with the implementation team and data collected earlier, we will use a targeted approach, driven by relevant domains of CFIR to collect data. We will further work with stakeholders to explore the link between specific domains and implementation strategies using the ERIC-CFIR matching tool [47]. Based on the preliminary stakeholder engagement, we have developed a logic model for the process evaluation (See Fig. 4).

Participants will include trial participants (patients and their carers), service providers (PCPs, lay counsellors and clinic supervisors), those involved in implementing the intervention (SINA administration and management staff), and other stakeholders relevant to wider adoption and sustainability (government sector primary care staff and policymakers). One-to-one interviews will be conducted with 10 to 15 patients and 2 to 3 focus groups with the clinic and SINA staff. Additionally, 2 focus groups will be conducted with implementation team members, including clinic staff and policymakers from government sector primary care facilities, to explore scale-up. Quantitative data guided by the CFIR domains will also be collected as part of the process evaluation which may include using routinely collected data (See Table 4).

**Table 4:** Process Evaluation Data Collection

Elements	Methods	Time points (months)	Content/indicators
**Fidelity**			
	Quantitative	1 & 6	Intervention Evaluating how staff and service users perceived adherence to essential elements of collaborative care, such as screening, coordination, providing evidence-based treatments, maintaining records, and utilising tracking, referral systems and ongoing training
**Reach**			
	Mixed	1 & 6	Intervention and treatment as usual Screening and treatment receipt rates, perceptions of service users regarding the accessibility of treatment, staff perceptions regarding the accessibility of resources, and training material
**Acceptability**			
	Mixed	1 & 6	Intervention Staff and service users’ perceptions about treatment acceptability, including screening, referrals, BA delivery and antidepressants
		1 & 6	Treatment as usual Staff and service users’ perceptions about the acceptability of treatment covering routine care
**Appropriateness**			
	Mixed	1 & 6	Intervention and treatment as usual Staff and service users’ perceptions regarding the compatibility of collaborative care while focusing on coordination, teaming up and access to resources
**Sustainability**			
	Mixed	6 & 12	Intervention Staff, service users, and policymakers’ engagement and perceptions to carry on staff and policymakers’ engagement
**Scale-up**			
	Qualitative	12	Intervention Implementation team and policymakers’ perspectives (including those involved in the trial and those not involved) on how to roll out to government settings, provincial and national levels

The interview, focus group guides, and quantitative data collection tools used to capture the above-provided information will be guided by applicable CFIR domains

Qualitative data will be analysed using a largely deductive thematic analysis approach. The computer management software NVivo will be used for managing qualitative data analysis. Quantitative data collected will be merged with the trial dataset. To elucidate any relationships, we will use mixed-effects regression models, enabling the exploration of both fixed and random effects (See statistical analysis).

### Economic evaluation

A within-trial cost-effectiveness analysis will be conducted to examine the value for money of CC over the trial period. This will be assessed from a healthcare perspective in the base case, with broader perspectives such as societal, also considered. Outcomes will be measured in both quality-adjusted life-years (QALYs) and disability-adjusted life-years (DALYs), generic health measures reflecting both morbidity and mortality. Resource use will be collected, and costs will be estimated by applying unit costs to resource use. Evidence on resource use will be presented. The EQ-5D-5L instrument will capture health-related quality of life for QALYs. At the same time, health conditions information will be collected to inform the application of disability weights from the Global Burden of Disease Study to capture disability for DALYs. Evidence on resource use associated with the intervention will be collected as part of the study. A modified Client Service Receipt Inventory will gather evidence on broader healthcare resource use and out-of-pocket costs. Information on household expenditures will be captured using a questionnaire to estimate catastrophic healthcare costs. Cost-effectiveness will be presented using incremental cost-effectiveness ratios, net health benefits, and net monetary benefits based on appropriate cost-effectiveness thresholds.

A secondary analysis will also consider the cost-effectiveness of the implementation activity. This will measure outcomes regarding the reach of depression care (as described previously) and costs from a healthcare perspective. Cost-effectiveness will again be presented using incremental cost-effectiveness ratios. Finally, a broader economic analysis will also consider the impact of the intervention on catastrophic healthcare costs.

## Discussion

Despite the availability of well-established, effective, evidence-based, and relatively low-cost treatments for depression, the mental health expertise and resources to provide these interventions are severely limited, leading to a large ‘mental health treatment gap’, with the majority of mental disorders remaining undetected and untreated, especially in LMICs [54, 55]. The CC with its elements of task shifting and shared care-based model has been proven to be both effective and acceptable approach to address this gap [56]. The current study aims to assess the effectiveness and implementation of CC model in 24 primary care clinics in Pakistan. To the best of our knowledge, no studies in LMICs in South Asia have evaluated implementation and effectiveness of CC.

The hybrid-II design will allow assessment of both the effectiveness (including cost-effectiveness) and implementation of the CC in SINA primary care clinics. Although it is acknowledged that the recruitment of study participants post randomisation of clinics risks potential selection bias. However, given the need to introduce changes to the intervention clinics, including staff training before in order to set-up CC may require up to 3 months. During this period, depression status and severity are likely to change, and it will also not be justifiable to withhold treatment for identified depression, precluding recruitment before the randomisation of clinics. To mitigate the resulting risk of selection bias in recruitment, we will attempt to maintain blinding of research staff who are recruiting participants to clinic allocation, ensure recruitment follows written protocols, and document reasons for excluding participants. Moreover, some study outcomes will include the whole clinic population through the use of aggregated anonymised electronic healthcare records, reducing the risk of bias from selective recruitment.

The strengths of the study include rapid translational gains, the development of effective implementation strategies through a co-design process involving relevant stakeholders, and timely information for decision-makers. The design also includes staff training and materials to promote OUC, which will improve provision of mental health care across SINA, the primary care provider organisation, irrespective of trial outcomes.

If CC is shown to be successfully implemented, effective, and cost-effective, it will provide health and economic benefits for people with depression presenting in primary care. It will strengthen primary care services, through a trained workforce that can recognise and manage the most common mental health problems, improved information management systems, and promotion of evidence-based care. It will also inform better integrated, less fragmented, person-centred care for mental health more widely, with strengthened connections between primary care and mental health services.

### Trial status

This is the current approved protocol version (V0.7), dated September 05, 2024. Recruitment began in May 2025 and is ongoing, expected to complete by March 31, 2026. Until 15th January 2026, 83% of the target recruitment has been achieved, which is 598/720 trial participants recruited.

### Data management

The data management process (including procedures for data quality, confidentiality, and security) will conform to the University of York’s Research Data Management policy to maintain data integrity, security, and privacy while sharing data with partners. A specified team from the ARK Foundation, Bangladesh will be involved in routine data management tasks such as identifying and resolving data inconsistencies or errors, monitoring for missing data and ensuring completeness, and performing quality checks to maintain the integrity and accuracy of collected data. All data shared among SPIRIT-D researchers will be shared through a secure platform. As a standard procedure, all data will be encrypted before sharing. All study files will be stored in accordance with GCP guidelines and UK GDPR and Data Protection Act 2018. Only study identification (ID) numbers will be used to identify participants on electronic documents containing quantitative and qualitative data. Interviews or focus group recordings will be deleted once the data analysis is completed. Once analysed and reported, the anonymous participant-level data set will be uploaded to the sponsor institutional repository website and made available on request.

### Programme Steering Committee

An independent Programme Steering Committee (PSC) has been set up, including 6 members with expertise on mental health and global health research, trials and statistics, health economics expertise, to provide an oversight and specialist advice. PSC meetings are being held twice in the first year of the programme and then annually thereafter.

### Data Monitoring Committee

An independent Data Monitoring Committee (DMC) has been set up including 5 members with expertise in trials, statistics, mental health and health systems and services in Pakistan. The role of the DMC is to act as the oversight body for the SPIRIT-D trial on behalf of the Sponsor (University of York) and the Funder (National Institute of Health Research – NIHR), with the purpose of protecting and serving SPIRIT-D participants, particularly in matters of safety and to support and advise the Chief Investigator in maintaining the validity and credibility of the trial, safeguarding participant interests, assessing the safety of interventions, and monitoring the overall conduct of the trial.

### Harms

Any serious adverse event (SAE) observed in trial participants will be promptly reported to the chief investigator within two calendar days by the research team. Subsequently, the chief investigator will compile and generate a comprehensive SAE report, adhering to local (Pakistan) standards (rule 8, clinical trial management, drug regulatory authority) for expedited reporting. This report will be submitted to the Pakistan National Pharmacovigilance Centre (Division of Drug Regulatory Authority Pakistan) and local and international ethical boards within five calendar days of the initial notification. Immediate referral to pertinent health and support services will be facilitated to ensure the provision of timely and appropriate medical care. Researchers who collect data at all follow-ups will note any adverse effects or events.

### Auditing

An internal audit by senior research management will ensure adherence to the study protocol, monitor compliance with regulatory requirements, and assess data integrity. This process will identify deviations or inconsistencies, enabling corrective actions.

### Ethics and dissemination

The ethics approvals have been obtained from the University of York Health Sciences Research Governance Committee and the National Bioethics Committee, Pakistan. Any protocol amendments will be duly communicated to both the ethics committees for their approval. We will obtain written informed consent from all trial participants for their involvement in study procedures and use of their anonymised data in analysis and write-up. We do not anticipate any significant risks to patients from the intervention, which is, in effect, part of current best practice. To ensure the safety of the researchers and the participants, we will follow the University of York’s and our partner’s safeguarding policies. We plan to disseminate findings through scientific publications and presentations at local and international conferences. Our research network links strongly with the public, the healthcare community, academic institutions, and policymakers. We aim to use those networks to disseminate or offer a national scale-up plan involving those stakeholders.

### Trial sponsors

The University of York are the sponsors for the SPiRiT-D trial.

## Supplementary Information

Supplementary Material 1.

## Data Availability

Not applicable.

## Abbreviations

BA: Behavioural Activation
BMI: Body mass index
BP: Blood pressure
CC: Collaborative care
CFIR: Consolidated Framework for Implementation Research
CSRI: Client Service Receipt Inventory
DALYs: Disability-adjusted life-years
GAD: Generalised anxiety disorder
HICs: High-income countries
HRQoL: Health-related quality of life
IT: Implementation team
IWI: International Wealth Index
LMICs: Low- and middle-income countries
PCP: Primary care physician
PHQ: Patient Health Questionnaire
QALYs: Quality-adjusted life-years
REAIM: Reach Effectiveness Adoption Implementation Maintenance
WHODAS: World Health Organization Disability Assessment Schedule
WHO: World Health Organization
